# Association between reduced white matter integrity in the corpus callosum and serotonin transporter gene DNA methylation in medication-naive patients with major depressive disorder

**DOI:** 10.1038/tp.2016.137

**Published:** 2016-08-09

**Authors:** E Won, S Choi, J Kang, A Kim, K-M Han, H S Chang, W S Tae, K R Son, S-H Joe, M-S Lee, B-J Ham

**Affiliations:** 1 Department of Psychiatry, Korea University Anam Hospital, Korea University College of Medicine, Seoul, Korea; 2 Department of Brain and Cognitive Engineering, Korea University, Seoul, Korea; 3 Department of Biomedical Science, Korea University, Seoul, Korea; 4 Department of Medical Bioscience, Graduate School, Soonchunhyang University, Bucheon, Korea; 5 Brain Convergence Research Center, Anam Hospital, Korea University Medical Center, Seoul, Korea; 6 Department of Radiology, Korea University Medical Center, Korea University College of Medicine, Seoul, Korea; 7 Department of Psychiatry, Korea University Guro Hospital, Korea University College of Medicine, Seoul, Korea

## Abstract

Previous evidence suggests that the serotonin transporter gene (SLC6A4) is associated with the structure of brain regions that are critically involved in dysfunctional limbic-cortical network activity associated with major depressive disorder (MDD). Diffusion tensor imaging (DTI) and tract-based spatial statistics were used to investigate changes in white matter integrity in patients with MDD compared with healthy controls. A possible association between structural alterations in white matter tracts and DNA methylation of the SLC6A4 promoter region was also assessed. Thirty-five medication-naive patients with MDD (mean age: 40.34, male/female: 10/25) and age, gender and education level matched 49 healthy controls (mean age: 41.12, male/female: 15/34) underwent DTI. SLC6A4 DNA methylation was also measured at five CpG sites of the promoter region, and the cell type used was whole-blood DNA. Patients with MDD had significantly lower fractional anisotropy (FA) values for the genu of the corpus callosum and body of the corpus callosum than that in healthy controls (family-wise error corrected, *P*<0.01). Significant inverse correlations were observed between SLC6A4 DNA methylation and FA (CpG3, Pearson's correlation: *r*=−0.493, *P*=0.003) and axial diffusivity (CpG3, Pearson's correlation: *r*=−0.478, *P*=0.004) values of the body of the corpus callosum in patients with MDD. These results contribute to evidence indicating an association between epigenetic gene regulation and structural brain alterations in depression. Moreover, we believe this is the first report of a correlation between DNA methylation of the SLC6A4 promoter region and white matter integrity in patients with MDD.

## Introduction

The pathophysiology of major depressive disorder (MDD) involves interactions between susceptible genotypes, chronic stress and an adverse developmental environment, which lead to alterations in the biochemistry, neuroplasticity and structure of the brain.^1^ Neuroimaging studies on depression have consistently identified neuroanatomical alterations in gray matter regions that participate in affect regulation.^2^ Given that white matter tracts connect various gray matter areas of the brain, many studies have also investigated possible alterations in white matter architecture and integrity in patients with MDD. Techniques using diffusion tensor imaging (DTI) and tract-based spatial statistics (TBSS) have made it possible to detect such alterations in a more sensitive and accurate manner.^3^ Previous DTI studies have found significant correlations between depression and altered integrity of white matter tracts that contribute to emotional regulation,^4^ such as the superior longitudinal fasciculus, corpus callosum, uncinate fasciculus, internal and external capsule, cingulum and anterior corona radiata.^1^

The heritability of MDD is estimated to range from 31 to 42%^5^ and the pathogenesis is thought to be significantly influenced by multiple genes, although genetic linkage and association studies have not identified specific strong and consistent MDD susceptibility genes.^6^ Altered serotonergic neurotransmission has repeatedly been implicated as a key contributor to the etiology of depression;^7^ therefore, genes that contribute to regulation of serotonin activity have been widely studied in relation to MDD. Since the serotonin transporter is responsible for serotonin reuptake at the terminals and cell bodies of serotonergic neurons, and since it is the primary molecular target for selective serotonin reuptake inhibitors,^8^ the serotonin transporter gene (SLC6A4) has been particularly well-studied. One of the polymorphic sites in the SLC6A4 is an insertion/deletion in the 5′-flanking promoter region (serotonin transporter-linked polymorphic region, 5-HTTLPR), which is associated with MDD-related phenotypes, including vulnerability to MDD^9^ and response to antidepressant treatment.^10^

Studies suggesting an important interaction between genetic and environmental factors in the pathophysiology of MDD are accumulating,^11^ with the majority reporting an interaction between 5-HTTLPR and stress in the development of depression.^12^ However, the variability in transcription accounted for by 5-HTTLPR is likely low to moderate, and epigenetic mechanisms have therefore been emphasized.^7^ Epigenetic regulation of SLC6A4 expression by DNA methylation or histone acetylation has been suggested as a mechanism for gene–environment interactions that contribute to MDD.^13^ Increased cytosine–guanine (CpG) methylation at SLC6A4 promoter regions, has been associated with lifetime history of depression,^14^ post-stroke depression^15^ and worsening clinical presentation of depression.^16^ In contrast, increased second trimester maternal depressed mood was associated with decreased maternal and infant SLC6A4 promoter methylation.^13^ A negative association has also been reported, with no difference in SLC6A4 promoter methylation levels being observed in unmedicated MDD patients and healthy controls.^17^

Numerous studies have reported that 5-HTTLPR is associated with brain structure, particularly regional gray matter volume or thickness^18^ and white matter integrity,^19^ which may be associated with the dysfunctional limbic-cortical network activity found in MDD.^3^ Recent studies have also reported that the status of SLC6A4 promoter region DNA methylation is associated with gray matter structure and function.^7, 20, 21^ However, only a few studies have demonstrated a direct correlation between epigenetic gene regulation and structural brain alterations associated with depression.^22^ Furthermore, previous studies have not examined the association between changes in white matter structure and SLC6A4 DNA methylation in patients with MDD.

In the present study, we used DTI and TBSS to investigate changes in white matter integrity of patients with MDD compared with healthy controls, and the possible association between structural alterations of white matter tracts and SLC6A4 promoter region DNA methylation status. First, we hypothesized that patients with MDD would exhibit altered integrity in white matter tracts that are related to cortico-limbic circuit alterations associated with depression. Second, we hypothesized that white matter tracts with decreased integrity in MDD patients would be associated with increased methylation of CpG sites at the SLC6A4 promoter region.

## Materials and methods

### Participants

We studied 35 medication-naive patients with MDD who had never taken antidepressants before, and 49 healthy controls. Patients were recruited from the outpatient psychiatric clinic of Korea University Anam Hospital, located in Seoul, Korea. Diagnosis was determined by a board-certified psychiatrist, according to the Diagnostic and Statistical Manual for Mental Disorders, 4th Edition, Text Revision (DSM-IV-TR), using the Korean version of the Structured Clinical Interview for DSM-IV (SCID-IV). Severity of depression was measured by the 17-item Hamilton Depression Rating Scale (HDRS) on the day of magnetic resonance imaging (MRI) acquisition. Patients with primary or comorbid psychiatric diagnoses other than MDD were excluded from the study, including personality disorders of clinical significance. Patients with lifetime exposure to DSM-IV dependence- or abuse-related drugs, other than nicotine, caffeine and social drinking of alcohol, were also excluded. Patients with serious or unstable medical illnesses or primary neurological illnesses, such as cerebrovascular disease, Parkinson's disease and epilepsy were also excluded. Two patients had a history of suicide attempt in the present depressive episode, one being drug intoxication and the other self-strangulation, but both attempts were not performed to an injurious degree. The other 33 patients did not have a history of suicide attempt in the present depressive episode. Also 18 patients confirmed to have a psychiatric family history, while the other 17 patients denied such a history. However, the absence of psychiatric family history in these patients could not be ascertained, as the family members were not assessed directly. Forty-nine healthy controls matched for age, gender and education level were recruited by advertisements in the community. Healthy controls were screened for major psychiatric histories, and no healthy controls had psychiatric disorders. Healthy participants who consumed alcohol more than to a social degree were also excluded. The age of participants in both groups ranged from 21 to 64 years. All participants were right-handed, as revealed by the Edinburgh Handedness Test, and were self-identified Koreans with ethnic origin ascertained by confirming the ethnicity of three generations of the patients' families. The protocol was approved by the ethics review board of Korea University College of Medicine, and signed informed consent was obtained from all participants according to the Declaration of Helsinki.

### Analysis of SLC6A4 DNA methylation

Peripheral blood samples were obtained from all participants to analyze SLC6A4 DNA methylation, and the cell type used was whole-blood DNA. The CpG-rich region of the promoter between −34 and −5 relative to the transcriptional start included 5 CpG sites (CpG1=−34, CpG2=−30, CpG3=−16, CpG4=−7, and CpG5=−5). The region selected covers from −66 bp upstream to the transcription start site of SLC6A4, which is a part of the CpG island located from −230 bp to +960 bp of the gene, and shows the highest level of CpG methylation throughout the CpG island (http://www.ncbi.nlm.nih.gov/epigenomics). This means that the screened region in this study could represent the methylation levels of the CpG island. In addition, the CpG island is the only methylated site in the whole SLC6A4 region in the brain. Thus, we considered this region to be suitable for testing the relationship between CpG methylation levels and SLC6A4 expression.

Methylation analyses of the SLC6A4 promoter region were conducted using the bisulfite pyrosequencing method. PCR and sequencing primers were designed using Pyrosequencing Assay Design Software v2.0 (Qiagen, Hilden, Germany). PCR reactions were performed in a volume of 20 μl with ⩾20 ng converted DNA, 2.5 μl 10 × Taq buffer, 5 U hot-start Taq polymerase (Enzynomics, Daejeon, Korea), 2 μl of each 2.5 mM dNTP mixture, 1 μl of 10 pmol per μl Primer-S and 1 μl of 10 pmol per μl biotinylated-Primer-As. The amplification was conducted according to the general guidelines of pyrosequencing: denaturing at 95 °C for 10 min, followed by 45 cycles at 95, 55  and 72 °C, each for 30 s, and a final extension at 72 °C for 5 min. The PCR reaction (2 μl) was confirmed by electrophoresis in a 2% agarose gel and visualized by ethidium bromide staining. Single-stranded DNA templates were prepared from 16 to 18 μl of biotinylated PCR products using streptavidin Sepharose HP beads (Amersham Biosciences, Uppsala, Sweden) following the PSQ 96 sample preparation guide for multichannel pipettes. Fifteen picomoles of the respective sequencing primer were added for analysis. Sequencing was performed on a PyroMark ID system with the Pyro Gold reagents kit (Qiagen), according to the manufacturer's instruction and without further optimization. The percentages of individual methylation at five CpG sites using the pyrosequencing method were calculated and analyzed.

### MRI acquisition

Diffusion data were acquired on a 3.0 T Siemens Trio whole-body imaging system (Siemens Medical Systems, Erlangen, Germany). DTIs were acquired using an echo-planar imaging sequence with the following parameters: repetition time: 6300 ms; echo time: 84 ms; field-of-view: 230 mm; 128 × 128 matrix; 3-mm slice thickness with no gap; voxel size 1.8 × 1.8 × 3.0 mm; diffusion directions=20; number of slices=50; *b*-values: 0 and 600 s mm^−2^; acceleration factor (iPAT-GRAPPA)=2 with 38 reference lines for phase encoding direction and 6/8-phase partial Fourier.

### MRI processing

We used the Functional MRI of the Brain (FMRIB) Software Library (FSL version 4.1.9, Oxford, UK, http://www.fmrib.ox.ac.uk/fsl/) for preprocessing of raw DTI data, and performed voxel-wise statistical analyses of the fractional anisotropy (FA) data using TBSS version 4.1.9.^23^ First, the raw data for each participant were software-corrected for Eddy current distortions and head movements. Using the Brain Extraction Tool (BET; Oxford Centre for Functional Magnetic Resonance Imaging of the Brain (FMRIB), Oxford, United Kingdom), a single binarized brain mask was generated in diffusion space, using ‘1' to represent inside the brain and ‘0' to represent outside the brain (*b*=0). Last, the diffusion tensor models were fitted to the corrected data of the FA images.

We checked the quality of the FA images to filter out any distorted data before performing TBSS analysis. However, there were no distorted data due to participant motion or scanner noise. FA images from each participant were aligned to the FMRIB58_FA template image and transformed into a 1 × 1 × 1 mm^3^ standard space (Montreal Neurological Institute 152 standard) using the FMRIB Non-linear Image Registration Tool (FNIRT). Non-linear transformed FA images were applied to the original FA image for each participant. An averaged FA image was then generated using the transformed FA images and extracted to create a mean FA skeleton image representing the centers of all white matter tracts. The FA skeleton image was set to a threshold of FA>0.2, to include the major white matter pathways.

**Figure 1:**
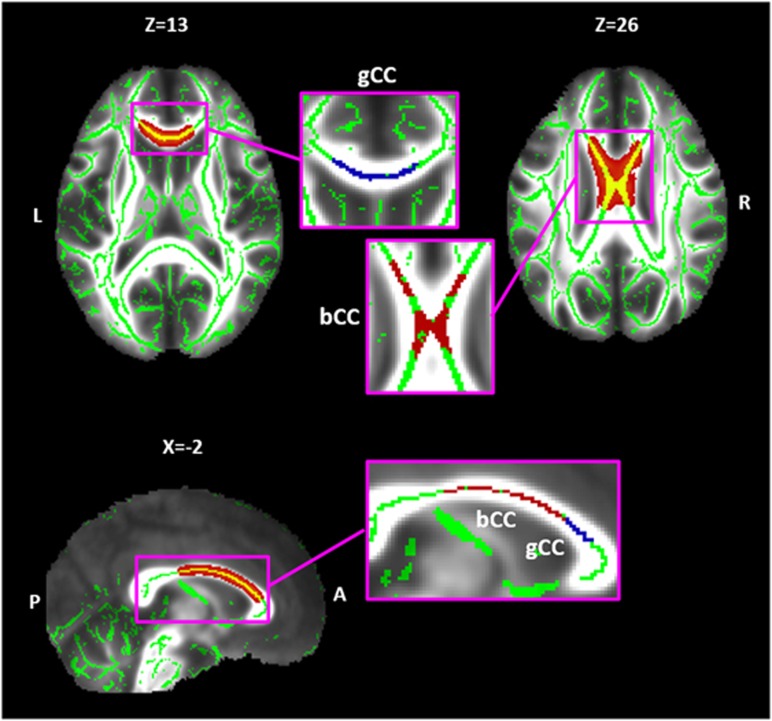
Results of tract-based spatial statistics (TBSS) analyses demonstrating significantly reduced fractional anisotropy (FA) values for the body of the corpus callosum (bCC) and genu of the corpus callosum (gCC) in medication-naive patients with major depressive disorder (MDD) (family-wise error corrected, *P*<0.01). Background images are the mean FA maps across all participants, with green voxels representing the mean FA skeleton images. Red and yellow voxels represent the white matter regions in which FA values were significantly lower in patients with MDD than in healthy controls. Enlarged images show the hand-drawn region-of-interest masks. Red areas represent the body of the corpus callosum and blue areas represent the genu of the corpus callosum.

Voxel-wise cross-subject statistics were applied to the skeletonized data for each participant using the FSL randomize program (University of Oxford, Oxford, United Kingdom). We performed a two-sample *t*-test for FA comparisons between patients with MDD and healthy controls. Permutation testing was performed with 5000 permutations using threshold-free cluster enhancement.^24^ The cluster size was always above 100 voxels and the level of significance was set at a family-wise error (FWE) corrected *P*<0.01 for multiple comparisons. The anatomical location of white matter tracts with significantly different FA values between patients with MDD and healthy controls were identified with the John Hopkins University (JHU) ICBM-DTI-81 white matter labels in FSL. Binary mask images were drawn from the identified regions using FSL, and were defined as the regions-of-interests (ROIs). Individual means of FA, axial diffusivity (AD) and radial diffusivity (RD) values were extracted from the ROIs (see Figure 1).

DTI expresses the character of the axon fiber using FA and other diffusivity values such as AD and RD. The FA index is sensitive to the presence and integrity of white matter fibers,^25^ and higher FA values represent either an increase in number and size of axon fibers, or a decrease in density of crossing fibers.^26^ Previous studies have suggested AD values to be sensitive to axonal pathologies and RD values to be sensitive to myelination,^27, 28, 29^ which may reflect white matter tissue microstructure that in turn affects functional connectivity within a neural circuit. Therefore, AD and RD values may complement FA values, and help to interpret potential underlying tissue microstructure alterations.^30^

### Statistical analyses

Differences in demographics between medication-naive patients with MDD and healthy controls were analyzed using two-sample *t*-tests for continuous variables (age, years of education and HDRS scores) and a *χ*^2^-test for gender. Analysis of covariance (ANCOVA) was performed to calculate differences in SLC6A4 promoter region methylation between patients with MDD and healthy controls, including age as a covariate, as an association between aging and DNA methylation has previously been reported.^31^ The alpha level was set at *P*<0.05/5=0.01 after a Bonferroni's correction for number of CpG sites (CpG1, CpG2, CpG3, CpG4 and CpG5).

The differences in AD and RD values of the ROIs between patients with MDD and healthy controls were analyzed using ANCOVA with age as a covariate, as age-related alterations in white matter microstructure have previously been reported.^32^ The ROIs were tested at *P*<0.05/2=0.025, after Bonferroni's corrections for number of ROIs.

A two-tailed Pearson's correlation was performed to analyze the correlations between the extracted FA, AD and RD values and SLC6A4 methylation, controlling for age, as an association between aging and DNA methylation,^31^ and alterations in white matter microstructure have previously been reported.^32^ Each ROI was separately tested at *P*<0.05/5=0.01 after Bonferroni's corrections for the five CpG sites. All statistical analyses were performed using SPSS version 12.0 (SPSS, Chicago, IL, USA). In addition, we analyzed the difference between the correlation coefficients of each diagnostic group, using Fisher's *r*-to-*z* transformation (two-tailed).

*Post hoc* statistical power calculations for the ANCOVA and comparison of correlation coefficients, were performed using the G*Power analysis program.^33^ The power calculations were based on the effect sizes which came from Cohen's *f* for ANCOVA and Cohen's *q* for correlation difference.^34^ Effect sizes were defined as small, medium or large according to the following criteria. For Cohen's *f*, 0.01⩽small effect size<0.25, 0.25⩽medium effect size<0.40, 0.40⩽large effect size. For Cohen's *q*, 0.01⩽small effect size<0.30, 0.30⩽medium effect size<0.50, 0.50⩽large effect size.^35^

## Results

### Demographic and clinical characteristics and SLC6A4 DNA methylation status

There were no significant differences in the demographic variables tested between patients with MDD and the healthy controls, with the predicted exception of HDRS scores (Table 1). The level of SLC6A4 DNA methylation was significantly higher at CpG2 in patients with MDD than in healthy controls (F(_1,81_)=8.365, *P*=0.005; Table 1), with medium effect size (Cohen's *f*=0.322, partial *η*^2^=0.094) as defined by Cohen.^35^

### TBSS analysis, AD and RD values

Medication-naive patients with MDD showed significantly lower FA values for the genu of the corpus callosum and body of the corpus callosum compared with healthy controls (FWE corrected, *P*=0.004; Table 2 and Figure 1). Therefore, the genu of the corpus callosum and body of the corpus callosum were defined as the ROIs.

**Figure 2:**
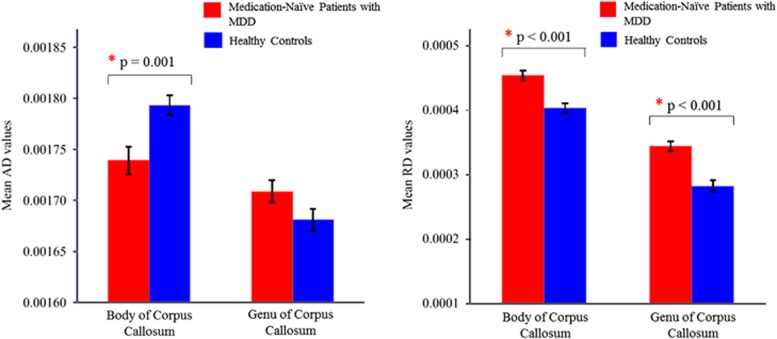
The mean axial diffusivity (AD) values for the body of the corpus callosum were significantly different between medication-naive patients with major depressive disorder (MDD) and healthy controls, with significantly decreased AD values of the body of the corpus callosum in patients with MDD. The mean radial diffusivity (RD) values for the body of the corpus callosum and genu of the corpus callosum were significantly different between patients with MDD and healthy controls, with patients showing significantly increased RD values. Bars represent s.e. '*' indicates significant *P-*values corrected for multiple comparisons using the Bonferroni's correction; *P*<0.05/2 (body of the corpus callosum, genu of the corpus callosum)=0.025.

AD values of the body of the corpus callosum were significantly different between the two groups. The MDD group demonstrated lower AD values than the healthy control group (F_(1,81)_=11.577, *P*=0.001), with medium effect size (Cohen's *f*= 0.322, *η*^2^=0.125). RD values of the body of the corpus callosum were also significantly different between the two groups. The MDD group demonstrated higher RD values than the healthy control group (F_(1,81)_=23.084, *P*<0.001), with large effect size (Cohen's *f*=0.53, *η*^2^=0.222). In addition, RD values of the genu of the corpus callosum were significantly different between the two groups. The MDD group demonstrated higher RD values compared with the healthy control group (F_(1,81)_ =27.793, *P*<0.001), with large effect size (Cohen's *f*=0.59, *η*^2^=0.255). These results are summarized in Table 3 and Figure 2.

### Correlations between FA, AD and RD values, and SLC6A4 DNA methylation status

**Figure 3:**
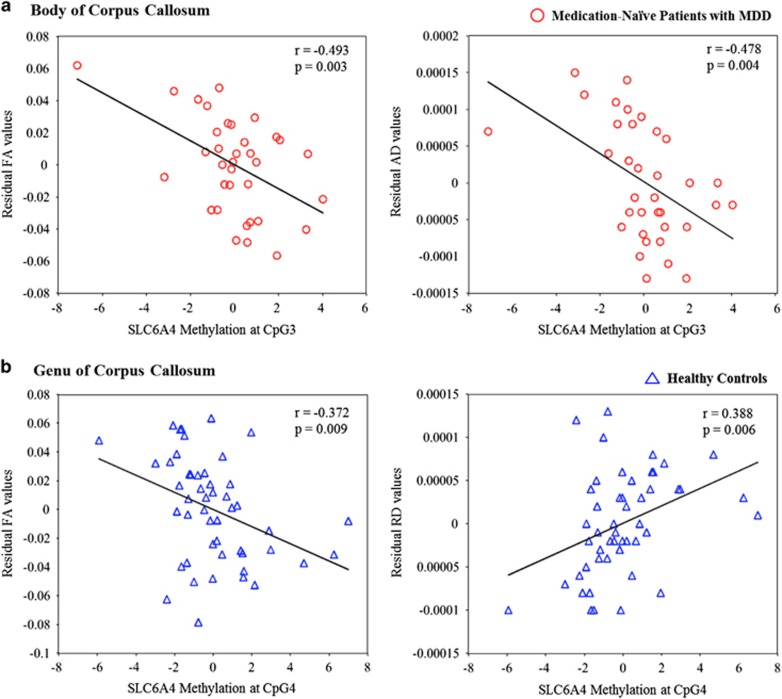
(**a**) Significant inverse correlations between fractional anisotropy (FA) values and axial diffusivity (AD) values for the body of the corpus callosum and serotonin transporter gene (SLC6A4) methylation at CpG3 were observed in medication-naive patients with MDD. (**b**) A significant inverse correlation between the FA values for the genu of the corpus callosum and SLC6A4 methylation at CpG4 was observed in healthy controls. In addition, a significant positive correlation between the RD values for the genu of the corpus callosum and SLC6A4 methylation at CpG4 was observed in healthy controls. MDD, major depressive disorder; RD, radial diffusivity.

A significant inverse correlation between the FA values of the body of the corpus callosum and SLC6A4 DNA methylation at CpG3 was observed for patients with MDD (Pearson's correlation: *r*=−0.493, *P*=0.003; Table 4 and Figure 3), in conjunction with a marginally significant inverse correlation at CpG4 (Pearson's correlation: *r*=−0.438, *P*=0.01; Table 4). A significant inverse correlation between the AD values of the body of the corpus callosum and SLC6A4 DNA methylation at CpG3 was also found in the MDD group (Pearson's correlation: *r*=−0.478, *P*=0.004; Table 4, Figure 3). In healthy controls, there were no significant correlations between any DTI measures of the body of the corpus callosum and SLC6A4 DNA methylation status.

For the genu of the corpus callosum, no significant correlations occurred in patients with MDD. However, a significant inverse correlation between the FA values of the genu of the corpus callosum and SLC6A4 DNA methylation at CpG4 was observed in healthy controls (Pearson's correlation: *r*=−0.372, *P*=0.009; Table 4 and Figure 3). In addition, a significant positive correlation between the RD values of the genu of the corpus callosum and SLC6A4 DNA methylation status at CpG4 occurred in the healthy control group (Pearson's correlation: *r*=0.388, *P*=0.006; Table 4 and Figure 3).

Patients with MDD showed significantly higher correlations between FA values of the body of the corpus callosum and SLC6A4 DNA methylation at CpG3 compared with healthy controls (FA: *z*=−2.16, *P*<0.05), with large effect size (Cohen's *q*=0.5). Also, patients with MDD showed significantly higher correlations between AD values of the body of the corpus callosum and SLC6A4 DNA methylation at CpG3 compared with healthy controls (*z*=−2.56, *P*<0.05), with medium effect size (Cohen's *q*=0.45). However, the correlations between FA and RD values of the genu of the corpus callosum and SLC6A4 DNA methylation at CpG4 in healthy controls were not significantly higher compared with patients with MDD (FA: *z*=0.57, *P*>0.1; RD: *z*=−1.04, *P*>0.1).

## Discussion

The present study found significantly lower FA and AD values and higher RD values of the body of the corpus callosum, as well as significantly lower FA values and higher RD values of the genu of the corpus callosum in patients with MDD. These findings indicate decreased integrity of the body of the corpus callosum and genu of the corpus callosum in these patients. Significantly elevated SLC6A4 DNA methylation was also observed in patients with MDD compared with that in healthy controls. Furthermore, we identified significant inverse correlations between DNA methylation status of the SLC6A4 promoter region and both FA and AD values of the body of the corpus callosum in patients with MDD. To the best of our knowledge, the present study is the first report of an association between brain white matter structural alterations and SLC6A4 DNA methylation status in patients with MDD.

The corpus callosum is the largest commissural white matter pathway connecting the hemispheres of the human brain, and is an essential component in brain lateralization and inter-hemispheric communication.^36^ The genu is the most rostral region of the corpus callosum and forms connections between prefrontal brain regions.^37^ The most caudal region of the corpus callosum is the splenium, and the body is the midsection between the genu and splenium. In general, the overall area of the corpus callosum increases during childhood and adolescence,^36^ and maturation of the corpus callosum corresponds to maturation of cognitive processes.^38^ The core physiological mechanism in this maturation process is axonal myelination,^39^ and disruption in the myelination trajectory can induce deficits in executive and daily functioning.^38^ Critical functions such as working memory, cognition and emotional processing can be negatively influenced by deficits of the corpus callosum^40^ which may predispose individuals to more severe depressive symptoms.^41^ Structural changes in the corpus callosum have been shown to induce altered hemispheric connectivity and impaired emotional and cognitive control, which are reported in patients with MDD.^42^ Treatment-naive young women with early-onset dysthymia,^43^ as well as adolescents and adults with early-onset MDD were reported to have a smaller corpus callosum compared with healthy controls.^38, 44^ DTI studies have reported lower FA values of the corpus callosum in treatment-naive young adults with a first episode of MDD,^45^ in middle-aged patients with MDD^4^ and in a group of MDD patients who were either treatment-naive or taking medication.^3^ FA deficits associated with lower AD and higher RD values in the corpus callosum have also been identified in patients with MDD.^46^ Also, a recent study has reported increased depression severity to be significantly related to reduced FA of the corpus callosum in patients with mild Alzheimer's disease.^47^ Our present findings are consistent with these previous studies reporting significant alterations in the corpus callosum of patients with depression.

We also observed a significant difference in DNA methylation at CpG2 of the SLC6A4 promoter region, with increased methylation in patients with MDD. This finding is consistent with a previous study that reported an association between increased SLC6A4 promoter methylation and susceptibility to major depression.^14^ Furthermore, we identified significant inverse correlations between FA and AD values of the body of the corpus callosum and SLC6A4 methylation at CpG3 in patients with MDD. Our results indicate a decrease in integrity of the body of the corpus callosum, which is associated with an increase in SLC6A4 promoter region methylation. This is similar to the results of a recent study which had reported increased SLC6A4 promoter methylation to be associated with brain structure, with smaller hippocampal volumes being associated with higher levels of methylation.^21^ The authors of this study stated that one of the underlying physiological mechanisms of how SLC6A4 methylation is associated with brain structure, may be the trophic actions of serotonin.^48^ We speculate the inverse correlation between methylation status and white matter integrity observed in our results, may also be explained by the role of SLC6A4 DNA methylation in regulating neuronal plasticity of the body of the corpus callosum, through alterations in serotonin activity.

Not only is serotonin involved in basic morphogenetic activities during brain development, such as modulation of neural cell proliferation, migration and differentiation, axonal guidance and synaptogenesis,^49^ but is also involved in adult neurogenesis.^50^ Chronic exposure to several forms of stress have been shown to alter chromatin structure in the brain, such as increases in DNA methylation or histone acetylation,^51^ and several studies have found associations between stress and altered promoter methylation levels of the SLC6A4 in particular.^52^ Also, epigenetic changes occur not only in the developing fetus, but also in individuals throughout the human life-span.^53^ High methylation density at the gene promoter region typically silences gene expression, so increased methylation of the SLC6A4 promoter region may result in a loss of gene functioning.^54^ The serotonin transporter is essential to determining the duration and intensity of serotonin communication with pre- and post-synaptic receptors,^55^ including the serotonin 1A receptor, which is implicated in synaptic function and plasticity.^48^ Therefore, decreased expression of SLC6A4 due to increased DNA methylation may decrease serotonin uptake and produce a deficiency in serotonin activity.^54^ Accordingly, SLC6A4 promoter methylation has been associated with lower *in vivo* measures of serotonin synthesis in specific brain areas.^56^ Given that alterations in serotonergic modulation can have unexpected effects on neuronal growth,^57^ brain regions with increased methylation of the SLC6A4 promoter region may exhibit dysfunctional brain morphology and physiology.^48^ As stress has repeatedly been associated with MDD, known to precipitate depressive episodes and influence the severity, duration and natural course of the disorder,^58^ patients with MDD are more likely to have been exposed to stress. Therefore, our results may represent an association between alterations in serotonin activity influenced by increased SLC6A4 promoter methylation levels due to stress exposure, and changes in white matter integrity in MDD.

We also observed a significant negative correlation between FA values of the genu of the corpus callosum and SLC6A4 methylation at CpG4, as well as a significant positive correlation between RD values of the genu of the corpus callosum and SLC6A4 methylation at CpG4 in healthy controls. These correlations were not significantly higher compared with patients with MDD, when the difference between correlation coefficients of each diagnostic group was analyzed.

To our knowledge, this is the first report on brain white matter structural changes and SLC6A4 promoter methylation in patients with MDD. Neurotrophic effects of antidepressants have been reported;^59^ therefore, only psychotropic medication-naive patients with MDD were included in the present study. Although our study has multiple strengths, there are also limitations to consider. There is no clear consensus regarding the appropriate number of CpG loci of the SLC6A4 promoter region for DTI analyses on SLC6A4 DNA methylation. However, the number of CpG loci in our study is relatively fewer compared with other recent studies,^13^ and may therefore affect the ability to detect a correlation between SLC6A4 DNA methylation status and white matter tract alterations in patients with MDD. Second, we did not assess psychosocial stressors such as childhood adversity and stressful life events. Stress has been shown to alter SLC6A4 promoter methylation levels,^52^ and white matter integrity has been reported to be sensitive to adverse experiences.^60^ Therefore, the influence of such environmental factors on our results is uncertain. However, as stated above, stress is known to precipitate depressive episodes and influence the natural course of the disorder.^58^ Therefore we considered patients with MDD more likely to have been exposed to stress compared with healthy controls. Third, the sample used to analyze SLC6A4 DNA methylation, was peripheral blood DNA. Although the majority of studies investigating DNA methylation patterns in humans have been performed on peripheral blood cells, such studies are considered to be constrained by access to tissue.^61^ As DNA methylation changes are tissue specific, it is suggested that the results of studies on epigenetic mechanisms need to be considered in the context of the cells that are assessed. Nevertheless, there is emerging evidence for the relevance of peripheral SLC6A4 methylation for brain processes,^21^ and DNA methylation at the SLC6A4 promoter is suggested not to be limited to peripheral tissues, but paralleled in the brain.^52^ Fourth, the age of participants in both groups ranged from 21 to 64 years, which can be considered high. However, our mean age (s.d.) for MDD patients was 40.34 (11.08), which is similar to previous DTI studies on major depression.^1^ Therefore, we considered the age range of our sample to be capable of representing MDD patients in general. Also, we considered concentrating on a certain age group would overly emphasize sample homogeneity, which may hinder generalizing our results. Furthermore, heavy alcohol consumption and smoking have been reported to affect white matter microstructure.^62, 63^ Although we had excluded participants who consumed alcohol more than to a social degree, the exact amount of alcohol consumption and proportion of smokers were not assessed, which we consider to be a limitation as the influence of such factors on our results is uncertain. Also, it has previously been suggested that a sample size of at least 50–100 patients is needed in imaging genetics studies, to report findings that are relatively consistent and achieve sufficient power.^64, 65^ For instance, Rasch *et al.*^66^ demonstrated that an ample total sample size for imaging genetics studies is at least 50 subjects to achieve sufficient statistical power. Therefore, our sample which included 35 patients and 49 healthy controls may be considered comparatively small. However, we consider the effect sizes for the ANCOVA and comparison of correlation coefficients of our study to be sufficient, as the difference of SLC6A4 methylation at CpG2 between groups and the difference between AD values of the body of the corpus callosum between groups showed medium effect size, and the difference between RD values of both the body and genu of the corpus callosum between groups showed large effect size. In addition, the significant difference in correlation coefficients between groups showed large effect size and medium effect size, for the correlations between FA values and AD values of the body of the corpus callosum and SLC6A4 DNA methylation at CpG3, respectively. Last, we used a non-isotropic voxel size (1.8 mm × 1.8 mm × 3.0 mm) in our study. Although non-isotropic voxels have been used for various reasons, such as to reduce scanning time in patients, and to compensate low signal-to-noise ratio of DTI and scanner's low intrinsic resolution in clinical studies, it has also been suggested that non-isotropic voxels may cause underestimation of FA in regions with crossing fibers.^67^ The results of this study were limited to the corpus callosum, which has one directional inter-hemispheric fibers. Therefore, we consider the crossing fiber bias for non-isotopic voxels, to not affect our significant findings on the corpus callosum. However, as the TBSS analysis was conducted on the whole brain, the estimation of FA of certain white matter tracts that contain crossing fibers may have been affected by voxel size.

We identified decreased integrity of the body of the corpus callosum and genu of the corpus callosum in patients with MDD compared with healthy controls, a significant difference in DNA methylation status of the SLC6A4 promoter region between the two groups, and a significant inverse correlation between SLC6A4 methylation status and white matter integrity of the body of the corpus callosum in MDD patients. The potential modulatory effects of gene–environment interactions on the structural integrity of white matter in depression should be assessed further in future investigations.

## Figures and Tables

**Table 1:** Demographic and clinical characteristics of drug naive patients with MDD and healthy controls

	*MDD patients (*n*=35)*	*Healthy controls (*n*=49)*	t*/*χ^*2*^*/F*	*df*	P*-value*
Age (years)	40.34 (11.08)	41.12 (14.11)	*t*=−0.283	81.2	0.786
Gender (male/female)	10/25	15/34	*χ*^2^=0.041	1	0.84
Education (years)	13 (3.02)	14.14 (3.06)	*t*=−1.696	82	0.094
HDRS-17^a^	20.2 (4.95)	2.18 (2.14)	*t*=20.222	43.11	<0.001^a^
Illness duration (months)	12.03 (24.55)	—	—	—	—
					
*SLC6A4 methylation (%)*					
CpG1	4.25 (1.76)	5.38 (2.14)	F=6.397	81	0.013
CpG2	2.45 (1.43)	1.61 (1.21)	F=8.365	81	0.005^b^
CpG3	6.90 (2.00)	6.10 (1.53)	F=4.702	81	0.033
CpG4	5.69 (1.96)	5.83 (2.24)	F=0.085	81	0.772
CpG5	1.75 (1.50)	1.59 (1.16)	F=0.31	81	0.579

Abbreviations: df, degree of freedom; HDRS-17, 17-item Hamilton Depression Rating Scale; MDD, major depressive disorder; SLC6A4, serotonin transporter gene.

Data are represented as mean (s.d.), unless otherwise indicated.

a Significance level for demographic and clinical characteristics. *P*<0.05.

b Significance level for SLC6A4 methylation was corrected for multiple comparisons using Bonferroni's correction; *P*<0.05/5 (CpG1, CpG2, CpG3, CpG4 and CpG5)=0.01.

**Table 2:** Regions showing significantly decreased FA values in drug naive patients with MDD compared with healthy controls

*Cluster size*	P*-value*^a^	*MNI peak coordinates (mm)*			*Anatomical region*
		X	Y	Z	
1872	0.004	−2	23	13	Genu of corpus callosum
					Body of corpus callosum

Abbreviations: FA, fractional anisotropy; MDD, major depressive disorder; MNI, Montreal Neurological Institute coordinates.

a Threshold-free cluster enhancement corrected *P*-value (family-wise comparisons, error corrected, *P*<0.01).

**Table 3:** AD and RD values of the bCC and gCC in drug naive MDD patients and healthy controls

	*MDD patients (*n*=35)*	*Healthy controls (*n*=49)*	*F*	P*-value*
*Axial diffusivity*				
gCC	0.001709 (1.08E−05)	0.001681 (1.09E−05)	3.204	0.077
bCC	0.001739 (1.32E−05)	0.001793 (9.43E−06)	11.577	0.001^a^

*Radial diffusivity*				
gCC	0.000345 (7.44E−06)	0.000283 (8.71E−06)	27.793	<0.001^a^
bCC	0.000454 (7.67E−05)	0.000404 (6.86E−06)	23.084	<0.001^a^

Abbreviations: AD, axial diffusivity; bCC, body of corpus callosum; gCC, genu of corpus callosum; MDD, major depressive disorder; RD, radial diffusivity.

Data are represented as mean (s.e.), unless otherwise indicated.

a Significance level was corrected for multiple comparisons using Bonferroni's correction; *P*<0.05/2 (BCC, GCC)=0.025.

**Table 4:** Results for Pearson's correlation analysis between FA, AD and RD values of the gCC and bCC and SLC6A4 methylation (CpG1, CpG2, CpG3, CpG4 and CpG5) in drug naive MDD patients and healthy controls

	*SLC6A4 methylation*				
	*CpG1*	*CpG2*	*CpG3*	*CpG4*	*CpG5*
*FA*					
MDD patients					
gCC	−0.068 (0.702)	−0.195 (0.27)	−0.28 (0.108)	−0.253 (0.15)	−0.132 (0.458)
bCC	−0.301 (0.084)	−0.255 (0.15)	−0.493 (0.003)^a^	−0.438 (0.01)^b^	−0.142 (0.422)
Healthy controls					
gCC	−0.191 (0.192)	−0.17 (0.249)	−0.203 (0.167)	−0.372 (0.009)^a^	−0.095 (0.519)
bCC	−0.022 (0.88)	−0.03 (0.840)	−0.042 (0.778)	−0.117 (0.428)	0.013 (0.931)

*AD*					
MDD patients					
gCC	−0.188 (0.288)	−0.315 (0.07)	−0.419 (0.14)	−0.361 (0.036)	−0.301 (0.084)
bCC	−0.225 (0.2)	−0.252 (0.151)	−0.478 (0.004)^a^	−0.341 (0.049)	−0.293 (0.092)
Healthy controls					
gCC	0.215 (0.143)	−0.139 (0.345)	−0.101 (0.493)	0.201 (0.170)	−0.042 (0.779)
bCC	0.024 (0.872)	0.012 (0.938)	0.068 (0.647)	0.082 (0.577)	−0.080 (0.587)

*RD*					
MDD patients					
gCC	0.034 (0.847)	0.124 (0.486)	0.190 (0.282)	0.168 (0.341)	0.042 (0.815)
bCC	0.237 (0.178)	0.156 (0.378)	0.307 (0.077)	0.294 (0.092)	0.021 (0.907)
Healthy controls					
gCC	0.200 (0.173)	0.153 (0.298)	0.175 (0.234)	0.388 (0.006)^a^	0.064 (0.664)
bCC	0.028 (0.850)	0.041 (0.780)	0.022 (0.883)	0.107 (0.470)	−0.041 (0.783)

Abbreviations: AD, axial diffusivity; bCC, body of corpus callosum; FA, fractional anisotropy; gCC, genu of corpus callosum; MDD, major depressive disorder; RD, radial diffusivity; SLC6A4, serotonin transporter gene.

All data are given as coefficient of Pearson's correlation controlling for age (*P-*value).

a Significance level for SLC6A4 methylation was corrected for multiple comparisons using Bonferroni's correction; *P*<0.05/5 (CpG1, CpG2, CpG3, CpG4 and CpG5)=0.01.

b Statistical significance: *P*=0.01.
